# Supplementary material for: Associations of IL-18 with Altered Cardiovascular Risk Profile in Psoriatic Arthritis and Ankylosing Spondylitis
Source: J Clin Med. 2022 Jan 30;11(3):766. doi: 10.3390/jcm11030766 (PMC8836492; doi:10.3390/jcm11030766)
Supplement: Supplementary file 1 [file jcm-11-00766-s001.zip › jcm-1534354-supplementary.pdf]

Supplementary Table S1. Extraarticular symptoms and frequency of HLA-B17 in AS and PsA patients.

**Measured parameters**	**AS *n*=94**	**PsAtotal *n*=61**
Dactylitis	3 (3.2%)	7 (11.5%)
Uveitis	34 (35%)	2 (3.3%)
Enthesitis	30 (30.9%)	8 (13.1)
HLA-B27	83 (88.3 %)	20 (32.8%)
